# Case Report: single low-dose of denosumab as a trigger of MRONJ development in a patient with osteoporosis after bisphosphonate therapy

**DOI:** 10.3389/froh.2024.1473049

**Published:** 2024-12-04

**Authors:** Dávid Száraz, Vojtěch Peřina, Jana Treglerová, Ctirad Macháček, Ondřej Zendulka, Petra Bořilová Linhartová

**Affiliations:** ^1^ Clinic of Maxillofacial Surgery, University Hospital Brno, Brno, Czechia; ^2^ Clinic of Maxillofacial Surgery, Faculty of Medicine, Masaryk University, Brno, Czechia; ^3^ Department of Pathology, University Hospital Brno, Brno, Czechia; ^4^ Department of Pathology, Faculty of Medicine, Masaryk University, Brno, Czechia; ^5^ Department of Pharmacology, Faculty of Medicine, Masaryk University, Brno, Czechia; ^6^ RECETOX, Faculty of Science, Masaryk University, Brno, Czechia

**Keywords:** osteonecrosis of the jaw, denosumab, statin, bisphosphonates, osteoporosis, MRONJ, case report, single dose

## Abstract

Both denosumab (DMB) and bisphosphonates (BPs), antiresorptive drugs (ARDs) used for the treatment of osteoporosis and oncological disorders, are known for their potential to cause medication-related osteonecrosis of the jaws (MRONJ). Besides ARDs, statins were recently associated with MRONJ development, especially in patients taking higher doses of statins for a longer period of time. Here, we report a case of a female patient with osteoporosis using statins and treated with alendronate for 3 years who rapidly developed MRONJ stage III after only a single low dose of DMB. After partial maxillectomy complete healing was observed without any recurrence. We performed a literature review of cases with MRONJ triggered by a single low dose of DMB, with or without previous application of other ARDs. Only six similar cases of patients who developed MRONJ after a single low dose of DMB following previous BP therapy have been reported so far. Besides these, literature reports one patient who developed MRONJ after a single dose of DMB following romosozumab treatment and five cases developing MRONJ after a single dose of DMB even without any previous ARD treatment. We suggest that before DMB therapy is initiated, all factors predisposing to MRONJ development should be considered.

## Introduction

Medication-related osteonecrosis of the jaws (MRONJ) is a disease caused by antiresorptive drugs (ARDs) alone or with concomitant immune modulators or antiangiogenic drugs but without the history of radiation therapy or metastatic disease of the jaws. It is characterized as a non-healing exposed bone of the maxillofacial region, or bone that can be probed through a fistula with a duration of at least eight weeks (1). Sometimes, the infection and inflammation are not limited only to the jaws, but can involve adjacent anatomical areas like the nose (2) or orbit (3, 4). In rare cases, the necrosis could directly affect the skull base, specifically the external auditory canal (5, 6).

ARDs, namely denosumab (DMB) and bisphosphonates (BPs), are the most common causative agents of MRONJ (1). Typically, ARDs are prescribed to patients with osteoporosis or oncologic patients. In this paper, however, we will only focus on osteoporosis patients, who (if treated with DMB), typically receive 60 mg DMB subcutaneously every six months. In the case of BPs, the dosage and frequency differ depending on the particular drug.

Literature is not consistent on the issue whether osteoporosis patients treated with DMB or BPs are at higher risk of developing MRONJ, with some studies claiming BP to bear higher risk of MRONJ development (7), while others state the opposite (8–10). According to Ruggiero et al. (1), the risk of MRONJ in patients treated with DMB ranges from 0.04% to 0.3%, depending on the duration of the therapy. In patients with osteoporosis treated with BPs, MRONJ risk is estimated to be ≤0.02% and ≤0.05% for intravenous and oral BPs, respectively (1).

There is no consensus on the magnitude of the association between the duration of treatment with BPs (and their cumulative dose) and the risk of MRONJ development in osteoporotic patients. Some authors state that the duration does not have a major impact on such risk (1), while others report an increased risk of MRONJ after 1 year of treatment (11).

It needs to be pointed out that ARDs are not exclusive for promoting MRONJ. Statins, i.e., 3-hydroxy-3-methylglutaryl coenzyme A reductase inhibitors, have been also suggested to be causative agent of MRONJ development; however, their role in this process is not fully understood. In patients on high-frequency intravenous BPs, statins might have an additive detrimental effect on the function and survival of monocytes and macrophages (especially at high doses). This may, in turn, increase the risk of infection in the jaws (12).

This paper presents the case of an osteoporosis patient who had been treated with alendronate and statins prior to receiving a single low dose of DMB. Three months later, she was diagnosed with stage III MRONJ. In addition, we provide a literature review, discussing possible risks associated with long-term BPs and statins.

## Case report

In June 2022, an 83-years-old female patient was referred to our Clinic of Maxillofacial Surgery, University Hospital Brno, with an exposed bone of the edentulous maxilla. The patient was wearing complete dentures for 3 years (since her last visit at the dentist). The dentures did not cause her any problems, but she felt that “the dentures did not fit properly”. After she had visited her dentist on May 2022 for bone exposure in the area +4,567 of the edentulous maxilla without purulent exudation, she was referred to our clinic.

The patient suffered from other comorbidities, including chronic obstructive pulmonary disease. In 2013, she underwent radical resection of adenocarcinoma of the left pulmonary lobe with dissection of the lymph nodes (T1N0M0) without any adjuvant therapy. In 2014, the patient suffered from phlebothrombosis of the right lower limb, and in 2015, a percutaneous coronary intervention with the insertion of a coronary stent was conducted due to STEMI myocardial infarction. In 2020, she had a total hip replacement of the left hip. Other diagnoses included Parkinson's disease, epilepsy, and osteoporosis.

In March 2022, she was prescribed a single dose of Prolia 60 mg (DMB) due to severe scintigraphy-confirmed progression of osteoporosis. The patient reported receiving only a single dose of DMB as antiresorptive therapy. Since this would be unusual, we contacted the patient's osteologist, who informed us that the patient had used Fosavance 70 mg (alendronate) in the past, taking one tablet per week from November 2017 until October 2020. Other medications she was using at the time of MRONJ development included Sorvasta 10 mg (rosuvastatin; one per day for 7 years), Godasal 100/50 mg (acetylsalicylic acid/glycine; one per day), Concor 2.5 mg (bisoprolol; one per day), Detralex 500 mg (micronised purified flavonoid fraction; one per day), Glepark 0.18 mg (pramipexole; twice per day), Ultibro Breezhaler 85/43 ug (glycopyrronium + indacaterol; once per day), Nolpaza 40 mg (pantoprazole; once per day), Milgamma N 40 mg/90 mg/0.25 ug (benfotiamine + pyridoxine + cyanocobalamin; twice per day) and calcium with vitamin D3 500 mg/800 IU (once per day). The patient did not report any allergies or smoking habits.

**Figure 1:**
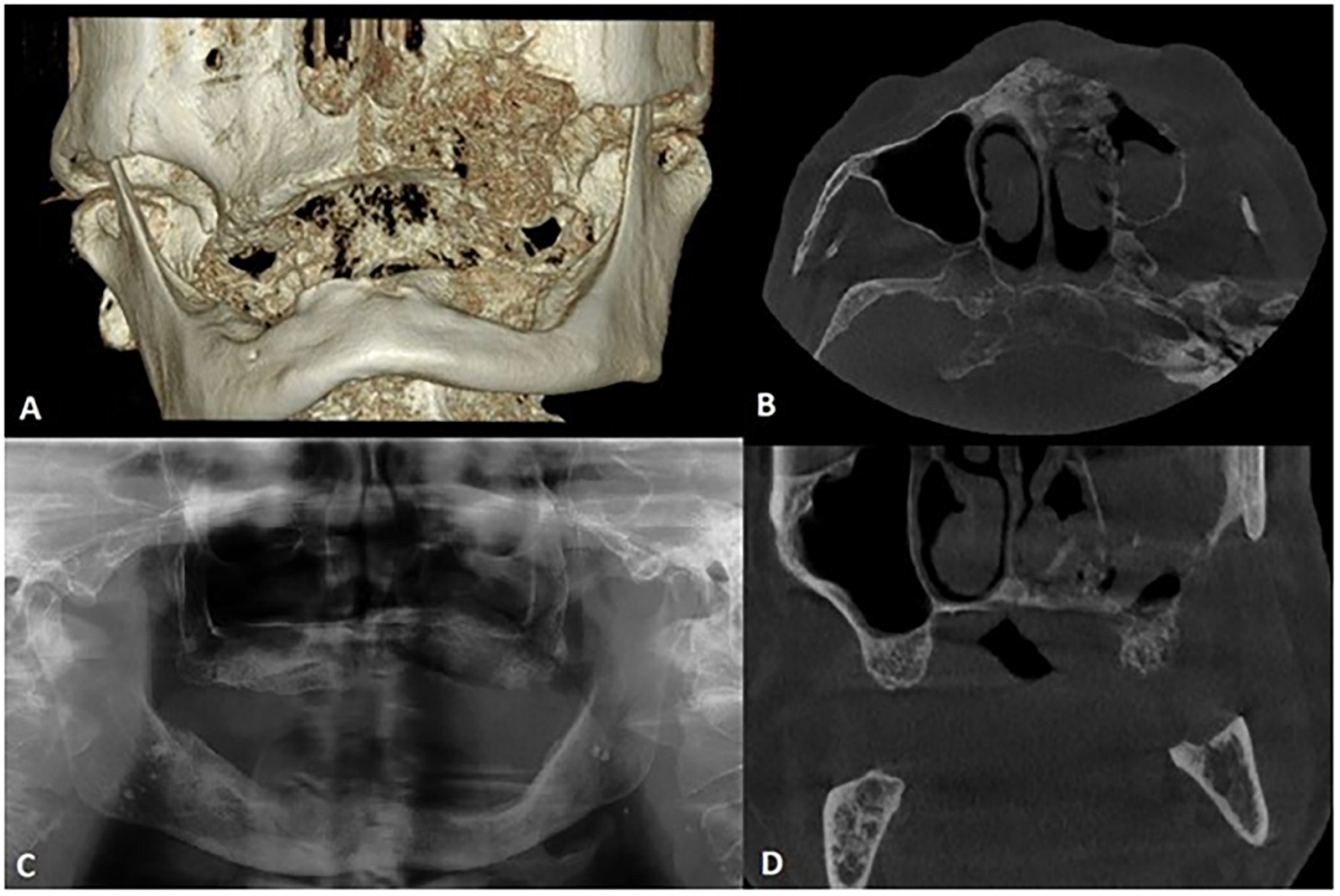
Pre-operative cone beam computed tomography and panoramic x-ray (both made with KaVo OP 3D Pro) of the presented patient. Images showing the extent of bone resorption of the left maxilla with infiltration of the left maxillary sinus. **(A)** 3D reconstruction, **(B)** Cross-section, **(C)** Panoramic x-ray, **(D)** Coronal-section.

Panoramic x-ray image (OPG, see Figure 1) revealed a typical picture of osteonecrosis with bone resorption in the area +4,567 of the maxilla. Additionally, a cone beam computed tomography revealed that the necrotic lesion of the bone involved the left side of the palate, including the floor of the nose and the anterior wall of the maxillary sinus reaching up to the infraorbital foramen. The necrosis involved the whole left half of the upper jaw, from the incisory area to the maxillary tuberosity (Figure 1). The final diagnosis was MRONJ of the left side of the maxilla (stage III) in June 2022, 3 months after the first dose of DMB.

**Figure 2:**
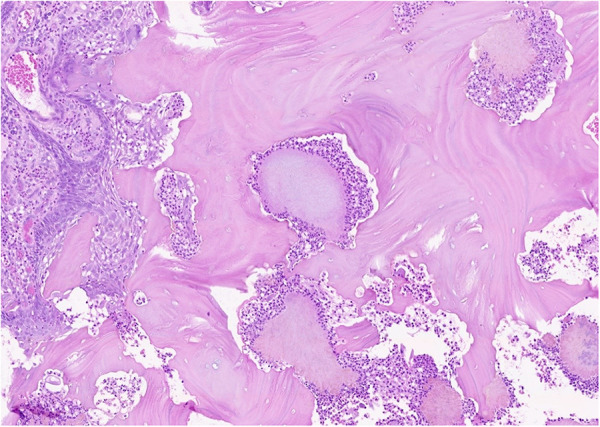
The necrotic bone of the maxilla from the presented case. Trabeculae of the necrotic bone with optically empty lacunae and local focuses of intertrabecular spaces with colonies of Actinomyces spp. surrounded by macrophages and neutrophils are apparent. The necrotic bone is superficially overgrown by squamous epithelium.

The patient was treated by partial resection of the maxilla. All the necrotic bone was removed by resection with safety margins in the healthy bone. The mucosa of the nasal cavity remained intact; however, the mucosa of the maxillary sinus showed inflammatory changes with granulation tissue and, hence, it was also removed. Histological examination confirmed osteonecrosis of the bone with reparatory changes; *Actinomyces* spp. were present (Figure 2). The inflammation of the removed mucosa of the maxillary sinus with granulation tissue was also confirmed. No malignant transformation was apparent in the specimens. The surgical procedure was preceded by pre-treatment with antibiotics, namely oral application of Amoksiklav 1 g (amoxicillin/clavulanic acid 875/125 mg; twice a day), 3 days prior to surgery. After the surgery, the patient was administered intravenous antibiotics (Amoksiklav, 1.2 g intravenously every 8 h) for a week and after her discharge from the clinic, she continued using antibiotics orally for another week.

**Figure 3:**
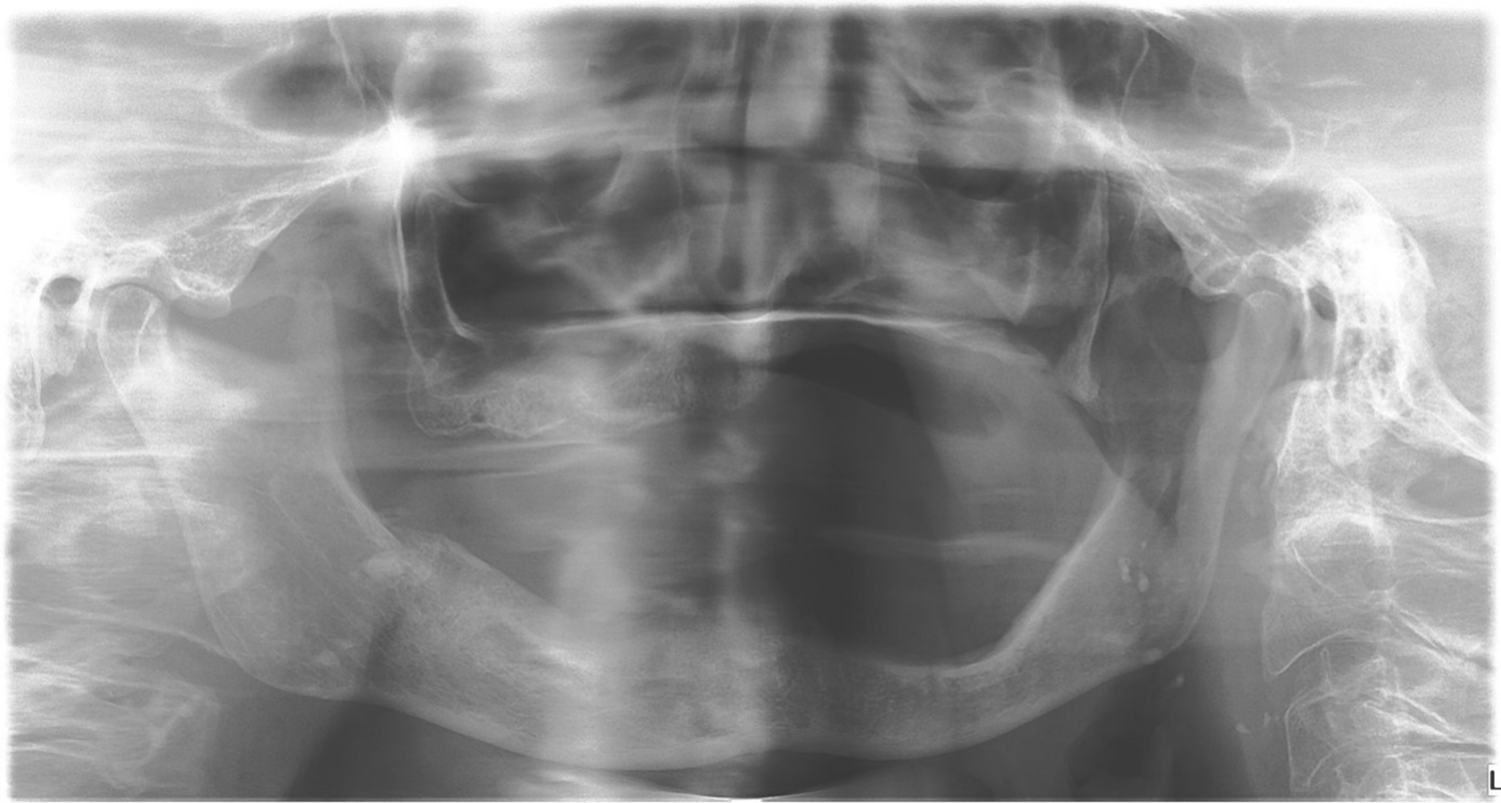
Presented patient's post-operative panoramic x-ray image (KaVo OP 3D Pro), 3 months after the surgery. No signs of residual MRONJ were observed.

**Figure 4:**
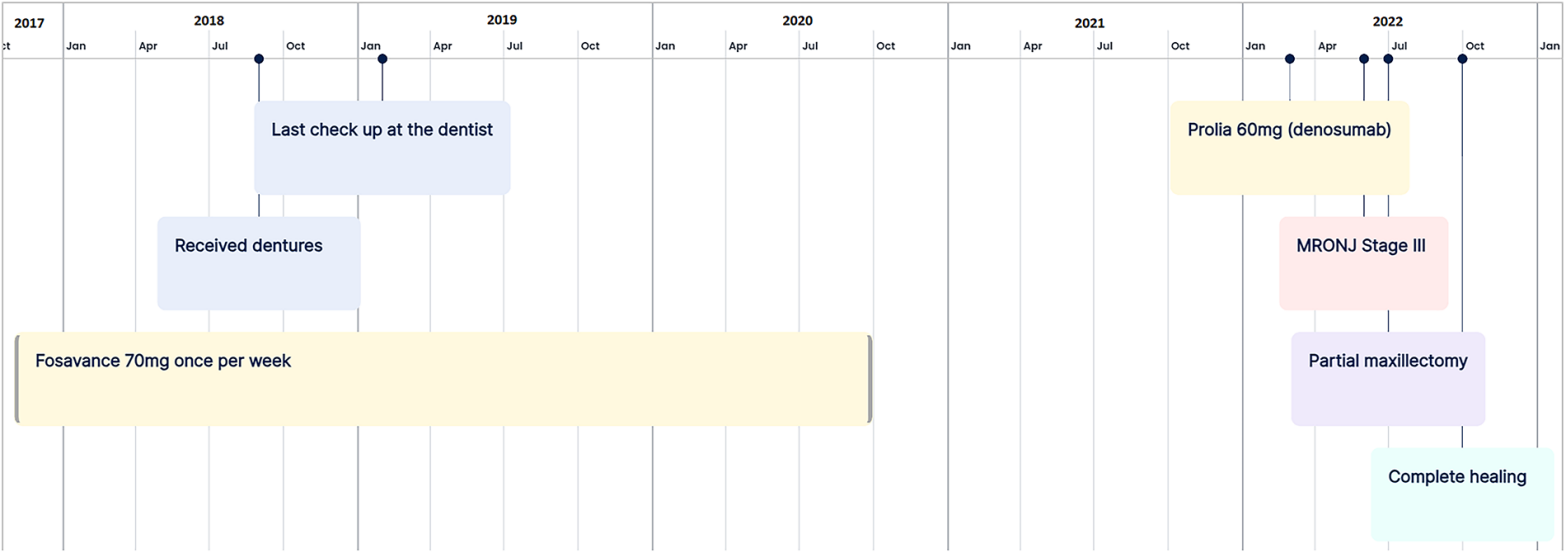
The timeline of important events in the presented case.

At the 3-month follow-up, complete healing was observed both clinically and on the OPG (Figure 3). The patient didn’t show any signs of recurrence or residual MRONJ. Unfortunately, no prosthetic rehabilitation will be possible due to a significant loss of bone; moreover, reconstruction surgery cannot be considered due to the patient's high age and comorbidities. Moreover, the patient found the intervention too radical and subsequently refused DMB and other ARDs. Figure 4 provides a concise summary of the presented case.

## Discussion

The incidence of MRONJ development in patients with osteoporosis treated with DMB is very low (ranging from 0.15% to less than 0.001% person-years of exposure) (13). Tofé et al. (14) reported the mean treatment time till MRONJ development with DMB to be approx. 31 months for low doses of DMB, while a recent multicentric study suggests a mean duration of 55 months (15). Marx stated in his book that MRONJ develops no sooner than after four applications of low-dose DMB, with an average number of eight doses before MRONJ development (16). However, several papers reported that the minimum number of DMB doses that can bring about MRONJ development can be lower. For example, Hoefert et al. (17) reported MRONJ stage III of the maxilla after 3 low doses of DMB at 6-month intervals in a 58 years old female, other papers reported MRONJ development even after a single dose of DMB (18–27).

A literature search for original articles, case reports, and case series with MRONJ development after a single low dose of DMB was conducted in PubMed and Scopus. The following combination of keywords was used for search: osteonecrosis AND jaw AND denosumab. Only reports on patients with osteoporosis who, besides other medication, received just a single low dose of DMB, were included in this review (Supplementary Table S1). In total, ten publications with twelve MRONJ cases were included (18–27). Except for 4 patients (20, 24, 26, 27), all of them were on some kind of medication possibly predisposing to MRONJ (BPs/statins/romosozumab). However, only limited information was available for some of these cases; moreover, it is possible that some risk factors have been omitted in some papers.

The number of DMB doses to MRONJ development in patients with osteoporosis is lower in patients previously treated by BPs (24) and it is suggested that patients transitioning from BPs to DMB are at higher risk of developing MRONJ than patients remaining on only on BPs (8, 24, 28). However, the issue remains controversial as the results were not confirmed by other studies. For example, Wick et al. (29) and Jung et al. (26) did not observe any difference in MRONJ occurrence between patients treated with DMB only or with BPs-DMB sequential therapy. Similarly, a recent study by Miller et al. (30) showed that none of the 1,413 patients in the cohort transitioning from BPs to DMB suffered from MRONJ. The controversy goes even further—Pautke et al. (10) reported that MRONJ developed significantly earlier in DMB-only treated patients than in those treated with BPs or BPs-DMB patients and that the success rate of treatment was lower in those treated with BPs than in the DMB or BPs-DMB group. The length of the “drug holiday” between BPs and DMB doesn't seem to have a significant impact on MRONJ occurrence, either (or at least not if it's shorter than 1 year) (7).

Our patient received only a single low dose of DMB and developed MRONJ within just three months afterward. It must be, however, taken into account that previously, she had been taking BPs for nearly 3 years in the past (with a cumulative dose around 1,000 defined daily doses). Although BPs had been terminated approximately 1.5 years before she received the single dose of DMB, the half-life for alendronate is, due to its irreversible binding to the bone, several years (16). Hence, it is plausible that MRONJ developed on the basis of a combined effect of BPs and DMB. Of the other contributing factors that may possibly play a role in the initiation of MRONJ development in our patient, the long-term rosuvastatin treatment may be considered.

Although rosuvastatin is considered very safe (31), several cases of statin-induced MRONJ without any previous antiresorptive therapy were published (32–35) (Supplementary Table S2). Also, two cases of DMB-induced MRONJ had statin treatment before DMB application (19, 25) (Supplementary Table S1). A recent cohort study hypothesized that there may be an interaction or relationship between DMB and hydrophilic statins (rosuvastatin, pravastatin) supporting MRONJ development (36).

Statins are discussed in relation to MRONJ etiopathogenesis (12) mainly because of their influence on bone metabolism (37, 38). Simvastatin, due to its protective effects on osteoblasts by acting on the mevalonate pathway and inhibiting osteoclastogenesis by blocking the RANKL pathway, is now considered to be a potential drug for osteoporosis treatment (39). However, rhabdomyolysis, a known adverse effect of statins (40) can be potentiated by DMB, with rapid onset seen even after a single dose of DMB (41). Several cases of mucosal ulcers were described as oral manifestations of adverse effects related to statins (42). Xerostomia, which is described in patients using statins, could be one of the underlying reasons (43, 44). This is similar to patients with Sjogren's syndrome, who typically suffer from dry mouth and often develop ulcers (45, 46). Therefore, it could be hypothesized that the effect of some statins on MRONJ development can be two-fold: (a) by damaging the mucosa, statins enable the oral bacteria to invade the bone and this, in combination with (b) direct involvement of statins in bone metabolism (the inhibition of osteoclastic differentiation and activity) can potentiate the negative effects.

On the other hand, short-term use of statins was shown to have many beneficial effects on oral health (47, 48). In fact, fluvastatin and atorvastatin have therapeutic effects on MRONJ and/or can act protectively (49–52). We can speculate that this ambivalent feature of statins could be explained by their possible dose-dependent pleiotropic effect: lower doses promote angiogenesis, while higher doses inhibit it (53). This seems to be plausible, since the cases of statin-induced MRONJ reported prolonged therapy, usually with higher doses of statins (Supplementary Table S2). Moreover, patients on long-term statin treatment often suffered from oral ulcers, which spontaneously healed after discontinuing statins (42). In our case, the patient also used rosuvastatin for several years, although in low doses.

It is not clear whether any of the other co-administered drugs can increase the risk of MRONJ; however, the effect of acetylsalicylic acid (ASA) and glycopyrronium in our patient cannot be excluded. ASA is known as an irreversible nonselective cyclooxygenase inhibitor with a dose-dependent effect. In our case, low-dose ASA was administered for secondary prevention of myocardial infarction. Recently, it was shown that ASA or its metabolites are able to reduce angiogenesis in multiple types of cancer (54). The mechanisms of the antiangiogenic effect of ASA are explained through inhibition of matrix metalloproteinases (55), influence on platelet activity (56), or effects of cyclooxygenases on endothelial cells (57). Most of the abovementioned effects of ASA are dose-dependent and low-dose ASA is, therefore, obviously not the main cause of MRONJ in our case. On the other hand, ASA can present an important piece in the puzzle of the total risk of MRONJ development. Glycopyrrolate, a peripheral competitive muscarinic receptor antagonist, should be also evaluated as an agent that shouldn't be omitted with respect to the risk of MRONJ. The decreased activity of salivary glands and xerostomia are some of the side effects of this drug.

Besides medication history, the higher age of the patient was the only other possible systemic risk factor to consider. The oncological status is not of particular interest, since the adenocarcinoma of the pulmonary globe was treated only surgically with complete remission. Regarding the local factors, dentures could be a causative factor; however, the patient did not report any significant problems with the dentures or any problems in the oral cavity prior to DMB application. On the other hand, we have to take into account that she did not visit her dentist for 3 years before the application of DMB, so discrepancies in the alignment of the denture with the alveolar process cannot be ruled out. In cases of MRONJ induced by BPs, the study of Hasegawa et al. (58) revealed that the duration to the onset of MRONJ was significantly shorter in patients with dentures than in those not wearing dentures. Given the high frequency of trauma caused by poorly fitting dentures (59–62), it is not surprising that dentures are also considered an important risk factor for MRONJ development (8, 58, 63). Hence, it is critical to regularly follow up the patients with dentures who are treated with DMB and all precautions should be made to prevent any trauma to the mucosa.

In all, the combination of BPs and DMB appears to be the most important causative factor in the rapid development of severe MRONJ in our patient. However, other additional factors may have played a role, such as the long-term rosuvastatin therapy, inappropriate denture adjustment, ASA, and glycopyrronium. All these factors may have predisposed the patient and created a susceptibility after which even a single low dose of DMB was enough to trigger MRONJ development. This suggests that patients at high risk of MRONJ should be carefully evaluated before switching to DMB administration in sequential therapy.

## Conclusion

Our paper presents a case of rapid MRONJ development within 3 months after administration of a single low dose of DMB in a patient who was previously receiving alendronate for 3 years. It is, therefore, likely that the cumulative dose of alendronate and its affinity to bone minerals created favorable circumstances for MRONJ development that was subsequently triggered by a single dose of DMB. Based on this case and the literature review performed during the preparation of this report, we can conclude that (i) patients should always be carefully examined before initiating treatment with DMB and/or switching from BPs to DMB and that (ii) all risk factors should be taken into account, including non-ARD medication (such as long-term statin therapy or ASA) and other predisposing factors that augment the possibility of MRONJ development. The preventive dental follow-up shouldn’t be underestimated, even in patients with total dentures.

## Data Availability

The original contributions presented in the study are included in the article/Supplementary Material, further inquiries can be directed to the corresponding author.
