# Supplementary material for: Case Report: single low-dose of denosumab as a trigger of MRONJ development in a patient with osteoporosis after bisphosphonate therapy
Source: Front Oral Health. 2024 Dec 4;5:1473049. doi: 10.3389/froh.2024.1473049 (PMC11652535; doi:10.3389/froh.2024.1473049)
Supplement: Supplementary file 1 [file Table1.docx]

Supplementary Material

**Supplementary Table S1**

Literature review of medication-related osteonecrosis of the jaw (MRONJ) triggered by a single low-dose of DMB (60 mg) in patients with osteoporosis

* one year “drug holiday” between risedronate and denosumab

N/A, non-applicable/specified, or unknown

DMB, denosumab

ARD, antiresorptive drug

| **Case** | **Age** | **Sex** | **ARD before DMB** | **Other medication** | **Medical history** | **Initiating factor** | **MRONJ location** | **MRONJ stage** | **MRONJ onset** | **Symptoms** | **Reference** |
| --- | --- | --- | --- | --- | --- | --- | --- | --- | --- | --- | --- |
| 1. | 75 | Female | Alendronate 70 mg/week for more than 3 years | N/A | Unclassified inflammatory bowel disease, previous glucocorticoid therapy, impaired renal function, chronic anemia, heterozygous b-thalassemia, mild to moderate iron overload | None | Mandible | N/A | 2 months after administration of DMB | MRONJ right lower jaw | Rachner et al. 2013 (18) |
| 2. | 58 | Male | No | Calcium (1 g/24 h) and vitamin D (800 IU/1 h), simvastatin | Myocardial infarctions (twice), hypertension | Dental extraction | Mandible | N/A | 5 months after DMB administration, 1 month after extraction | Pain, bone exposure | Neuprez et al. 2014 (19) |
| 3. | 84 | Female | No | None | Hypertension | Placement of dental implants | Mandible | N/A | 3 weeks after the placement of dental implants and approximately 23 weeks after DMB administration | Pain, bone exposure, peri-implant suppuration | Garcia Garcia et al. 2015 (20) |
| 4. | 65 | Female | Risedronate for a total of 4 years followed by annual zoledronic acid injections for a period of two years with the last dose being roughly 1 year prior to the extraction | N/A | Hypertension, gastroesophageal reflux disease, iron deficiency anemia, and rheumatoid arthritis | Dental extraction | Mandible, ulceration of the soft palate | N/A | 3 weeks after extraction and 4 weeks after DMB administration | Cutaneous fistula in the left submandibular region, spontaneous necrosis of the right side of the soft palate, ultimately developed acute respiratory distress syndrome | Qaisi et al. 2016 (21) |
| 5. | 78 | Female | Ibandronate for 43 months | N/A | N/A | Dental extraction | Mandible | Stage I | N/A | Bone exposure | Bagan et al. 2016 (22) |
| 6. | 77 | Female | Risedronate for 84 months | N/A | N/A | Dental extraction | Mandible | Stage I | N/A | Bone exposure | Bagan et al. 2016 (22) |
| 7. | N/A | N/A | Romosozumab for 12 months | N/A | N/A | Dental extraction | N/A | N/A | N/A, DMB administered 1 month after extraction | N/A | Cosman et al. 2016 (23) |
| 8. | 81 | N/A | Alendronate for 55 months | N/A | N/A | None | Mandible | N/A | N/A | N/A | Yarom et al. 2018 (24) |
| 9. | N/A | N/A | N/A | N/A | N/A | N/A | N/A | N/A | N/A | N/A | Yarom et al. 2018 (24) |
| 10. | 59 | Female | No | Acetylsalicylic acid (100 mg/24 h); atorvastatin (20 mg/ 24 h); hydrochlorothiazide (25 mg/24 h); 1 syringe/183 days), calciferol (25.000 UI/2.5 ml/ twice a week) | Poliomyelitis, meningitis, hypothyroidism due to hemithyreoidectomy due to microcalcifications, breast cancer, and received chemotherapy with partial removal of the breast, scoliosis, hypocalcemia, bronchial asthma, C5-C6 disc protrusion, microcytic anemia, right, carpal tunnel inversion, two strokes, heavy smoker | Dental extraction | Mandible | Stage II | 4 months after extraction, 3 months after DMB administration | Pain, bone exposur, with mucosa ulceration | Bujaldón-Rodríguez et al. 2019 (25) |
| 11. | 54 | Female | No | N/A | N/A | Dental extraction | Mandible | Stage II | 4 months after extraction | MRONJ on the lingual side of the mandible | Jung et al. 2022 (26) |
| 12. | 75 | Female | No | N/A | N/A | Dental extraction | Mandible | N/A | 2 months before root canal treatment and subsequent extraction | Inflammation, later fistula | Papadimitriou and Melakopoulos 2023 (27) |
| 13. | 83 | Female | Aledronate 70 mg/week for 3 years | Rosuvastatin (10 mg/24h) for 7 years, acetylsalicylic acid/glycine (100 mg/50 mg/24h), bisoprolol (2.5 mg/24h), micronised purified flavonoid fraction (500 mg/24 h), pramipexole (0.18 mg/12 h), glycopyrronium + indacaterol (85 mg/43 mg/24 h), pantoprazole (40 mg/24 h), benfotiamine + pyridoxine + cyanocobalamin (40 mg/90 mg/0.25 ug/12 h), calcium + vitamin D3 (500 mg/800 IU/24 h) | Chronic obstructive pulmonary disease, radical resection of adenocarcinoma of the left pulmonary lobe (T1N0M0) with dissection of the lymph nodes without any adjuvant therapy, phlebothrombosis of the right lower limb, percutaneous coronary intervention with the insertion of a coronary stent was conducted due to ST elevation myocardial infarction, total hip replacement of the left hip, Parkinson’s disease, epilepsy (without any recent paroxysms). | Most likely trauma caused by dentures | Maxilla | Stage III | 3 months after DMB application | Bone exposure | Current study |
