# Supplementary material for: Case Report: single low-dose of denosumab as a trigger of MRONJ development in a patient with osteoporosis after bisphosphonate therapy
Source: Front Oral Health. 2024 Dec 4;5:1473049. doi: 10.3389/froh.2024.1473049 (PMC11652535; doi:10.3389/froh.2024.1473049)
Supplement: Supplementary file 2 [file Table2.docx]

Supplementary Material

**Supplementary Table S2**

Literature review of cases with medication-related osteonecrosis of the jaws (MRONJ) on statin therapy, who are not users of antiresorptive drugs

* In the article by Nordi and Ghazali two of three patients reported diabetes mellitus, not specified which ones (33).

N/A, non-applicable/specified, or unknown

| **Case** | **Age** | **Sex** | **Statin** | **Other medication** | **Medical history** | **Triggering factor** | **MRONJ Location** | **MRONJ stage** | **Symptoms** | **Therapy** | **Duration for healing** | **Reference** |
| --- | --- | --- | --- | --- | --- | --- | --- | --- | --- | --- | --- | --- |
| 1 | 53 | Male | Atorvastatin 80 mg | N/A | * | Dental extraction | Mandible | N/A | Pain, bone exposure | Surgical debridement and primary wound closure | 4 months | Nordi and Ghazali 2022 (33) |
| 2 | 71 | Female | Atorvastatin 10 mg | N/A | * | Dental extraction | Maxilla | N/A | Pain, bone exposure | Surgical debridement and primary wound closure | 4 months | Nordi and Ghazali 2022 (33) |
| 3 | 61 | Male | Atorvastatin 10 mg | N/A | * | Dental extraction | Mandible | N/A | Pain, bone exposure | Surgical debridement and primary wound closure | 4 months | Nordi and Ghazali 2022 (33) |
| 4 | 48 | Female | Simvastatin 40 mg, received for 10 years | N/A | N/A | Dental extraction | Mandible | N/A | Pain, Swelling, Purulent drainage | Surgical debridement, application of platelet-rich fibrin, primary wound closure. | N/A | Samierad et al. 2021 (34) |
| 5 | N/A | N/A | Simvastatin 40 mg, received for more than 20 years | N/A | N/A | Oral surgery | Maxilla | N/A | Bone exposure | Debridement, antibiotic therapy | N/A | Giladi et al. 2020 (35) |
| 6 | N/A | N/A | Simvastatin 40 mg, received for more than 20 years | N/A | N/A | Oral surgery | Maxilla | N/A | Bone exposure | Debridement, antibiotic therapy | N/A | Giladi et al. 2020 (35) |
| 7 | 70 | Female | Rosuvastatin | Amitriptyline, celecoxib, fluticasone, hydrochlorthiazide, loratadine, andtemporarily amoxicillin | Osteoarthritis of the hip, previously received local hip steroid injections | None | Maxilla | III | Bleeding | Local rinsing with chlorhexidine | 6 months | Aghaloo et al. 2017 (32) |
